# Efficacy and prognosis of dapagliflozin in the treatment of patients with acute myocardial infarction complicated with type 2 diabetes in Xining area

**DOI:** 10.3389/fcvm.2025.1500978

**Published:** 2025-02-12

**Authors:** Jinping Chai, Delian Li, Yanmin Liu, Xiaoling Su

**Affiliations:** Department of Cardiovascular Medicine, Qinghai Provincial People’s Hospital, Xining, China

**Keywords:** acute myocardial infarction, type 2 diabetes, dapagliflozin, prognosis, complications

## Abstract

**Background:**

The acute myocardial infarction (AMI) is a prevalent and severe cardiovascular disease, characterized by its sudden onset, high mortality rate, and unfavorable prognosis. The presence of type 2 diabetes not only signifies a chronic metabolic disorder, but also serves as a catalyst for various cardiovascular and cerebrovascular ailments such as coronary heart disease and stroke. Xining is situated in a region of middle to high altitude and due to its unique geographical environment, coupled with the population's limited health awareness, unequal medical standards and other factors, there remain some AMI patients who are difficult to diagnose early on. The objective of this study is to investigate the efficacy and prognosis of dapagliflozin in patients with acute myocardial infarction complicated by type 2 diabetes in the Xining region.

**Method:**

analysis on January 1, 2018 to January 1, 2020, in Qinghai province people's hospital of cardiovascular internal medicine hospital treatment of 245 cases of acute myocardial infarction combined the clinical data of patients with type 2 diabetes. The patients were divided into dapagliflozin group and control group according to whether they took dapagliflozin during hospitalization. The basic data, laboratory examination indicators and long-term prognosis of the two groups were observed. Follow-up deadline is December 31, 2023, at the end of follow-up, including the primary endpoint and the secondary endpoint.

**Results:**

245 patients were included in this study, age 34–94, the average age (61–11), 200 cases (81.63%) of men, women, 45 cases (18.37%), dapagliflozin group of men 92 cases (77.97%) and control group, 108 cases (85.04%). Two groups of patients' age, gender, diabetes duration, merge disease, echocardiogram and blood biochemical indexes, had no statistical difference (*P* > 0.05). There were no significant differences in the number of coronary artery lesions, treatment regimens, cardiovascular and hypoglycemic drugs between the two groups (*P* > 0.05). However, up to dapagliflozin group of patients after discharge significantly lower than the control group, the incidence of cardiovascular adverse events at dapagliflozin group of 4 cases of heart failure and cardiovascular death in 1 case and control group in heart failure 13 cases, 10 cases of cardiovascular death, cerebral hemorrhage 2 cases died. KaplanMeier survival analysis showed that the primary endpoint of survival was significantly higher in the dapagliflozin group than in the control group (*P* < 0.05). In addition, the overall survival rate of the dapagliflozin group was significantly higher than that of the control group, and the difference was statistically significant (*P* < 0.05).

**Conclusions:**

Dapagliflozin is safe and reliable in the treatment of patients with acute myocardial infarction and type 2 diabetes, and can effectively reduce the incidence of cardiovascular events and improve the overall survival rate of patients.

## Introduction

Acute myocardial infarction (AMI) is a prevalent acute and severe cardiovascular diseases, characterized by its sudden onset, high mortality rate, and unfavorable prognosis (1–3). With the rapid advancement of medical technology, the level of early diagnosis and effective treatment of AMI has been improved (4, 5). However, Xining is situated in a region of middle to high altitude and due to its unique geographical environment, coupled with the population's limited health awareness, unequal medical standards and other factors, there remain some AMI patients who are difficult to diagnose early on. This results in delayed or suboptimal treatment as well as inadequate postoperative patient management which ultimately leads to heart failure, sudden cardiac death or other serious complications. The presence of type 2 diabetes not only signifies a chronic metabolic disorder, but also serves as a catalyst for various cardiovascular and cerebrovascular ailments such as coronary heart disease and stroke (6, 7).

The literature (8–10) has consistently demonstrated that diabetes is an independent risk factor for poor prognosis in cardiovascular disease. Moreover, a significant majority of acute myocardial infarction (AMI) patients often present with comorbid type 2 diabetes, which not only poses challenges in clinical management but also significantly escalates cardiovascular mortality among such individuals. Then, the efficacy in the prevention and treatment of acute myocardial infarction (AMI) combined with type 2 diabetes is suboptimal, failing to effectively reduce the incidence of cardiovascular events and improve patient prognosis (11). However, the introduction of a novel sodium-glucose co-transporter 2 (SGLT-2) inhibitor called dapagliflozin has sparked high expectations among clinicians regarding its potential for treating patients with type 2 diabetes who have experienced acute myocardial infarction (AMI) (12).

Currently, there is a scarcity of domestic research on the significant enhancement and improvement in quality of life as well as long-term prognosis for AMI patients with type 2 diabetes mellitus through dapagliflozin treatment, and there exists an insufficiency of evidence-based medical foundation (13). The objective of this study is to investigate the impact of dapagliflozin on the prognosis of patients with acute myocardial infarction (AMI) complicated by type 2 diabetes, aiming to provide robust evidence supporting dapagliflozin as the preferred choice for clinical management in such patients.

## Methods

### Research object

A total of 415 patients with AMI combined with type 2 diabetes who were hospitalized in the Department of Cardiovascular Disease of Qinghai Provincial People's Hospital from January 2018 to January 2020 were analyzed, and 245 patients qualified for the study were incorporated into the group after screening by inclusion and exclusion criteria. Inclusion criteria: (1) Patients who fulfill the diagnostic criteria for acute myocardial infarction (AMI) as outlined in the fourth edition of the Global Definition of Myocardial Infarction (14), including typical symptoms and signs of myocardial ischemia, dynamic changes in electrocardiogram, elevated markers of myocardial injury, etc.; (2) Patients diagnosed with type 2 diabetes according to the Chinese Guidelines for the Prevention and Treatment of Type 2 Diabetes (2020 Edition) (15) and currently receiving hypoglycemic medication for glycemic control. Exclusion criteria: (1) Patients with lost follow-up and poor compliance will be excluded from the study. (2) Patients with severe liver and renal insufficiency will not be included in the study population. (3) Patients who exhibit intolerance to the drugs used in this research will be excluded. (4) Patients presenting severe respiratory tract infection, urinary tract infection, fever, or other systemic infections combined will not be considered for participation. (5) Individuals diagnosed with other types of diabetes will be excluded from the study group. (6) Tumor patients are not eligible for inclusion in this research project. (7) Patients lacking complete clinical data will also be excluded. The present study adhered to the principles of medical ethics, and the acquisition of medical records was authorized by our hospital's Ethics Committee, thereby waiving the need for informed consent.

### The index of observation and its categorization

The observation measures included patients’ demographic information, admission blood pressure, comorbidities (such as hypertension, atrial fibrillation, stroke), blood biochemical markers (fasting blood glucose, glycated hemoglobin, liver and kidney function indicators), echocardiographic parameters (left ventricular ejection fraction, left ventricular end-diastolic volume), cardiac functional grade (Killip grade), treatment plan, and medication regimen. Patients were categorized into two groups: the dapagliflozin group (patients who had taken dapagliflozin either in the past or during hospitalization) and the control group (patients taking other hypoglycemic agents excluding dapagliflozin).

### Follow-up and study endpoint

The enrolled patients were monitored through telephone follow-up, outpatient visits or inpatient records. The primary endpoint was defined as the composite outcome of cardiac mortality, heart failure events, and cerebrovascular insult stroke. The secondary endpoint was defined as death resulting from cardiac and cerebrovascular causes, excluding other factors. Adverse events associated with daglipzin included hypoglycemia, hypovolemia, diabetic ketoacidosis, acute kidney injury, and genitourinary infection. The follow-up period concluded on December 31, 2023, with a median duration of 42 months.

### Statistical approach

The statistical software SPSS 25.0 was utilized for conducting data analysis. *T*-test was employed to compare groups when dealing with measurement data following a normal distribution. For measurement data that did not follow a normal distribution, they were represented as *M(Q25,Q75)*, and the Mann-Whitney test was used for group comparisons. Count data were expressed as rates or component ratios, and group comparisons were conducted using either *χ*^2^ test or Fisher exact probability method. Survival analysis was performed using the Kaplan-Meier method, and the Log-rank test was employed to compare survival rates between groups. A significance level of *P* < 0.05 indicated statistical difference.

## Results

### The clinical data of dapagliflozin group and control group were compared

A total of 245 patients, ranging in age from 34 to 94 years with an average age of (61 ± 11) years, were enrolled in this study. Among them, there were 200 males (81.63%) and 45 females (18.37%). In the daglipzin group, there were 92 males (77.97%), while the control group consisted of 102 males (85.04%). No statistically significant differences were observed between the two groups in terms of clinical data including age, gender, diabetes duration, comorbidities, blood biochemical parameters and echocardiographic indices (*P* > 0.05), as presented in Table 1.

**Table 1:** Comparison of clinical data between dapagliflozin group and control group.

Index	Dapagliflozin Group (*n* = 118)	Control group (*n* = 127)	*t/X^2^/Z*	*P*
Age (years)	63 ± 12	61 ± 11	1.261	0.209
Male (cases)	92	102	0.205	0.651
Duration of diabetes (years)	10 (7,13)	10 (8,13)	−0.239	0.811
Smoking background (cases)	58	52	1.666	0.197
Hypertension (cases)	59	59	0.308	0.579
Atrial fibrillation (cases)	40	41	0.072	0.788
Historical background of stroke (cases)	16	13	0.647	0.421
SBP (mmHg)	124 (116,144)	124 (111,145)	−0.679	0.497
DBP (mmHg)	79 (70,90)	79 (70,88)	−0.431	0.667
HbA1c (%)	6.39 (5.57,8.98)	6.13 (5.93,6.76)	−0.195	0.845
FPG (mmol/L)	8.90 (5.57,14.33)	8.40 (7.56,9.44)	−0.384	0.701
Cr (*μ*mol/L)	79 (71,96)	77 (70,88)	−1.579	0.114
UA (μmol/L)	316 (250,408)	299 (258,352)	−1.448	0.148
LDL-C (mmol/L)	2.49 (1.95,3.23)	2.53 (1.96,3.29)	−0.010	0.992
HDL-C (mmol/L)	1.16 (0.90,1.44)	1.25 (1.10,1.44)	−1.729	0.084
TC (mmol/L)	3.75 (3.22,4.44)	3.54 (3.12,4.07)	−1.444	0.149
TG (mmol/L)	1.50 (1.01,2.12)	1.65 (1.30,2.23)	−1.749	0.080
BNP (pg/ml)	587 (404,838)	580 (377,749)	−1.197	0.231
LVEF (%)	53 (50,60)	55 (51,60)	−1.404	0.160
LVEDV (ml)	111 (102,123)	112 (102,122)	−0.586	0.558

SBP refers to systolic blood pressure, while DBP stands for diastolic blood pressure. HbA1c represents glycosylated hemoglobin, FPG denotes fasting plasma glucose, Cr indicates creatinine levels, UA signifies uric acid levels, LDL-C represents low-density lipoprotein cholesterol, HDL-C stands for high-density lipoprotein cholesterol, TC refers to total cholesterol levels and TG represents triglyceride levels. BNP is an abbreviation for B-type natriuretic peptide and LVEF denotes left ventricular ejection fraction. Lastly, LVEDV stands for left ventricular end-diastolic volume.

### The cardiac function classification, treatment plan and incidence of endpoint events were compared between dapagliflozin group and control group

The number of coronary artery lesions, treatment regimen, cardiovascular and other hypoglycemic drugs did not differ significantly between the dapagliflozin group and the control group (*P* > 0.05). However, the incidence rates for both primary endpoint and secondary endpoint were lower in the dapagliflozin group compared to the control group with statistically significant differences (*P* < 0.05). Please refer to Table 2.

**Table 2:** Comparison of cardiac function, treatment plan and drug use between dapagliflozin group and control group.

	Dapagliflozin Group (*n* = 118)	Control group (*n* = 127)	*X^2^*	*P*
Killip classification				
Class I	96	100	0.469	0.791
Class Ⅱ	16	18		
Class Ⅲ	6	9		
Utilization of medication				
Metformin	72	76	0.035	0.851
Inhibitors of Alpha-glucosidase	12	15	0.168	0.682
Insulin	57	64	0.107	0.744
Agonists of the GLP-1 receptor	11	10	0.164	0.686
DDP-IV Inhibitor	10	12	0.071	0.790
Sulfonylureas	4	4		1.000*
Thiazolidinedione	3	5		0.724*
ARNI	16	21	0.423	0.516
ACEI/ARB	96	102	0.043	0.836
β-blocker	105	114	0.039	0.843
Calcium channel blockers	14	17	0.128	0.720
Loop diuretics	38	42	0.021	0.885
Spironolactone	44	54	0.698	0.404
Number of diseased vessels				
Single branch	15	17	1.522	0.467
Double branch	25	35		
Three branches	78	75		
Available Treatment Options				
PCI	110	117	0.108	0.743
Non-surgical intervention	8	10		
Follow-up outcomes				
Primary endpoint			15.187	0.001
Heart failure	4	13		
Cardiovascular death	1	10		
Stroke		2		
Secondary endpoint				
Respiratory failure	1	5		
Gastrointestinal bleeding	2			
Acute liver failure		1		
Renal failure		1		

Note: *Fisher's exact test. GLP-1, glucagon-like peptide-1; DDP-Ⅳ, dipeptidyl peptidase-4; ARNI, angiotensin receptor neprilysin inhibitor; ACEI/ARB, angiotensin-converting enzyme inhibitor/angiotensin II receptor blocker; PCI, percutaneous coronary intervention.

### Comparison of follow-up status and overall survival analysis between dagliferen group and control group of patients

**Figure 1:**
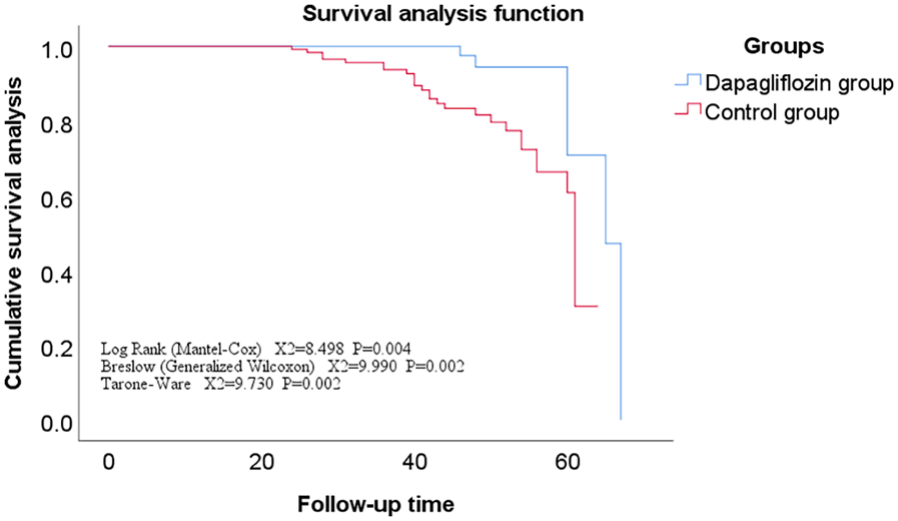
Survival curve of the main endpoints events in the dapagliflozin group versus the control group.

**Figure 2:**
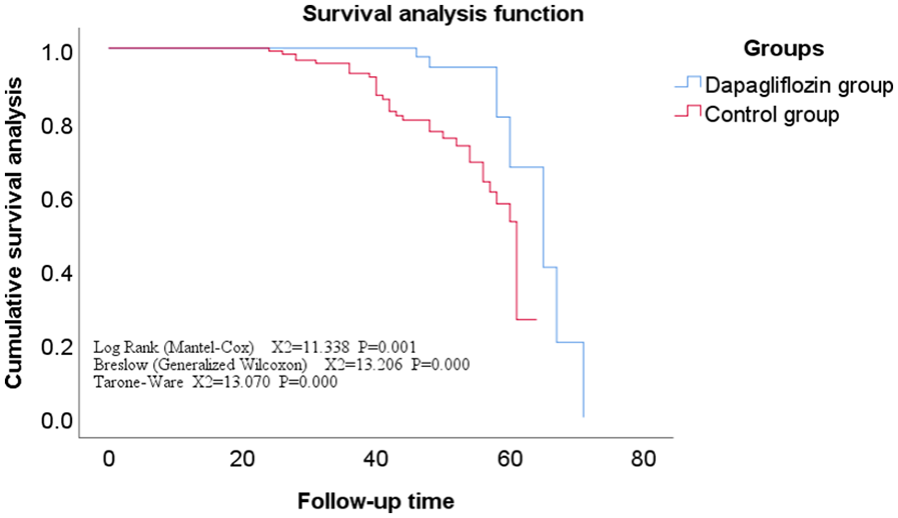
Survival curves for secondary endpoints in the dapagliflozin group versus the control group.

**Figure 3:**
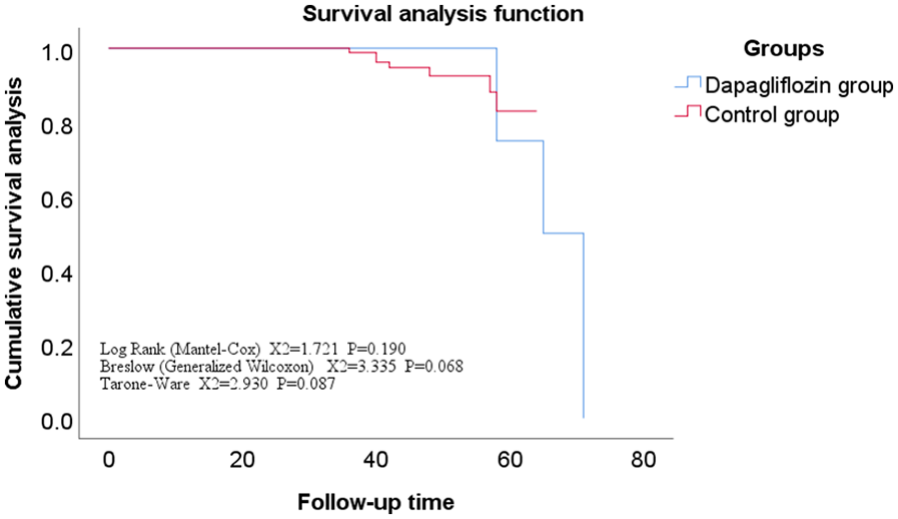
Overall survival curve for dapagliflozin group vs. control group.

All enrolled patients completed follow-up, ranging from 12 to 71 months, with a mean of 42 ± 11 months. The Kaplan-Meier survival analysis showed no statistically significant difference in the incidence rates of secondary endpoints between the Dapagliflozin group and the control group (*P* > 0.05). However, the incidence rate of the primary endpoint was significantly lower in the Dapagliflozin group than in the control group, with a statistically significant difference (*P* < 0.05). Furthermore, the overall survival rate was significantly higher in the Dapagliflozin group than in the control group, with a statistically significant difference (*P* < 0.05), as shown in Figures 1–3.

### Comparison of adverse events between dagliferen group and control group patients

During the hospitalization period, a total of 5 drug-related adverse reactions occurred in both groups. Among them, 1 case of hypoglycemia and 1 case of urinary tract infection occurred in the group treated with dapagliflozin; 2 cases of hypoglycemia and 1 case of urinary tract infection occurred in the control group. No other serious drug-related adverse reactions occurred in either group. There was no statistically significant difference in the incidence of drug-related adverse reactions between the two groups (*P* > 0.05).

## Discussion

Xining, located at an average altitude of 2,300 meters, is known as the eastern gateway to the Tibetan Plateau and is one of the high-altitude cities in the world. Xining has a continental plateau semi-arid climate or a high-altitude cold and temperate climate, characterized by low pressure, low oxygen, low temperature, and low humidity. Several studies have shown (16, 17), due to its unique climate environment, the incidence and mortality rate of cardiovascular diseases in Xining are lower than those in the plains, especially the incidence and mortality rate of AMI are lower than the national level. However, some studies have shown (18), the high-altitude environment can produce transient or long-term effects on blood pressure, heart rate, cardiac structure and function, and the long-term residence altitude is positively correlated with blood pressure level. However, some scholars conducted a population-based epidemiological investigation on the incidence of AMI in Xining area over the past 10 years and pointed out that due to the gradual improvement of people's living standards in the area, the incidence of AMI and diabetes has shown an upward trend (19–21). Type 2 diabetes is a metabolic disease characterized by chronic hyperglycemia, and AMI is a common complication of type 2 diabetes. Moreover, when type 2 diabetes patients suffer from AMI, they usually have multiple vessel lesions, which makes them more prone to heart failure, ultimately leading to poor treatment outcomes, high mortality rate, and poor prognosis. Studies at home and abroad have shown that (22–25) dapagliflozin, a novel anti-diabetic drug, not only has the function of lowering blood sugar but also improves left ventricular remodeling. Its safety and reliability for AMI patients with type 2 diabetes are better than those of other similar anti-diabetic drugs, and it can effectively improve and enhance the cardiac function and quality of life of these patients.

The results of this study indicate that men account for a larger proportion of patients with AMI and type 2 diabetes, and that high-risk factors such as smoking, hypertension, and atrial fibrillation account for about 50% of the total. Secondly, the study included patients with Killip functional classification of II or III, which accounted for about 18.6%–21.3% of the total. These patients had varying degrees of congestive heart failure during their hospital stay. However, after being treated with dapagliflozin and other cardiovascular drugs, AMI patients with type 2 diabetes showed a significant improvement in their heart failure symptoms compared to those treated with other therapies. This is consistent with the findings of many domestic scholars, who have found that dapagliflozin can reduce the volume of interstitial fluid without affecting blood volume, thereby significantly reducing the degree of pulmonary edema in patients (26). Moreover, the findings of this study indicate that instances of heart failure occurred in both the Dapagliflozin group and the control group following discharge. Nevertheless, the incidence rates of heart failure, cardiovascular mortality, and other related cardiovascular events were significantly lower in the Dapagliflozin cohort compared to the control group. This is corroborated by international research (9, 27), which demonstrates that Dapagliflozin can substantially diminish the risk of cardiovascular death or hospitalization due to heart failure in patients with type 2 diabetes by 17%. Furthermore, among patients with acute myocardial infarction (AMI), Dapagliflozin has been shown to reduce the likelihood of major adverse cardiovascular events by approximately 16%, thereby markedly enhancing cardiovascular outcomes for individuals with type 2 diabetes and AMI. There is also evidence from several domestic studies (28) that dapagliflozin has similar findings in Chinese patients with AMI and type 2 diabetes as in foreign studies. Therefore, early use of dapagliflozin can not only better control blood sugar and blood pressure in patients with AMI and type 2 diabetes, but also improve their cardiac function, reverse left ventricular remodeling, and reduce the incidence of cardiovascular adverse events (29, 30). Additionally, other studies have shown (31) that regardless of whether they have type 2 diabetes, dapagliflozin used in combination with conventional anti-heart failure drugs can significantly improve the cardiac function of AMI patients, reduce the incidence of cardiovascular adverse events, and improve the long-term prognosis of patients. At the same time, the results of this study show that there were a total of 5 drug-related adverse reactions in the dapagliflozin group and the control group during hospitalization, but there was no significant difference in the incidence of adverse reactions between the two groups, and no other serious drug-related adverse reactions occurred.The findings of numerous domestic and international studies (32–34) have also corroborated the aforementioned theory derived from this study, namely that dapagliflozin exhibits adverse reactions similar to other hypoglycemic drugs, including urinary tract infection, hypoglycemia, ketoacidosis, and hypotension. However, no study has demonstrated a significantly higher incidence of adverse reactions with dapagliflozin compared to other antidiabetic medications. Therefore, in terms of drug safety, dapagliflozin is deemed as safe and reliable as other hypoglycemic drugs (35). Nevertheless, in order to mitigate the occurrence of adverse reactions associated with dapagliflozin usage, the dosage of dapagliflozin should be strictly controlled according to the specific conditions of the patients, and patients are advised to increase their water intake, maintain perineal hygiene practices diligently and engage in regular physical activity (36).

In conclusion, the combination of dapagliflozin with conventional anti-heart failure drugs is not only safe but also effective in reducing cardiovascular adverse events and improving the long-term prognosis of patients with AMI and type 2 diabetes. However, this study has several limitations, such as being a single-center retrospective study with a limited sample size, incomplete clinical observation indicators, and failure to conduct a multivariate analysis of confounding factors. Therefore, in order to obtain more reliable research results, we plan to conduct a large-sample, multicenter prospective study in the future.

## Data Availability

The original contributions presented in the study are included in the article/Supplementary Material, further inquiries can be directed to the corresponding author/s.
